# Exploration of Scaffolds from Natural Products with Antiplasmodial Activities, Currently Registered Antimalarial Drugs and Public Malarial Screen Data

**DOI:** 10.3390/molecules21010104

**Published:** 2016-01-16

**Authors:** Samuel Egieyeh, James Syce, Alan Christoffels, Sarel F. Malan

**Affiliations:** 1 South African Medical Research Council Bioinformatics Unit, South African National Bioinformatics Institute, University of the Western Cape, Cape Town 7535, South Africa; segieyeh@sanbi.ac.za (S.E.); alan@sanbi.ac.za (A.C.); 2 School of Pharmacy, University of the Western Cape, Cape Town 7535, South Africa; jsyce@uwc.ac.za

**Keywords:** natural products, antimalarial drugs, scaffold diversity, scaffold tree

## Abstract

In light of current resistance to antimalarial drugs, there is a need to discover new classes of antimalarial agents with unique mechanisms of action. Identification of unique scaffolds from natural products with *in vitro* antiplasmodial activities may be the starting point for such new classes of antimalarial agents. We therefore conducted scaffold diversity and comparison analysis of natural products with *in vitro* antiplasmodial activities (NAA), currently registered antimalarial drugs (CRAD) and malaria screen data from Medicine for Malaria Ventures (MMV). The scaffold diversity analyses on the three datasets were performed using scaffold counts and cumulative scaffold frequency plots. Scaffolds from the NAA were compared to those from CRAD and MMV. A Scaffold Tree was also generated for each of the datasets and the scaffold diversity of NAA was found to be higher than that of MMV. Among the NAA compounds, we identified unique scaffolds that were not contained in any of the other compound datasets. These scaffolds from NAA also possess desirable drug-like properties making them ideal starting points for antimalarial drug design considerations. The Scaffold Tree showed the preponderance of ring systems in NAA and identified virtual scaffolds, which may be potential bioactive compounds.

## 1. Introduction

There is considerable interest to address the challenge of discovering new and novel small-molecule pharmaceuticals in view of the minimal success of compounds in combinatorial libraries to yield blockbuster drugs [1]. Natural products however remain a rich source of biologically active substances [2,3,4,5] and have made significant contribution to the antimalarial arsenal [6]. Based on this and the recent significant contribution of artemisinin, a natural product, to the antimalarial armoury, natural products with antiplasmodial activities (NAA) reported in literature [7,8] may be potential resources for discovery or design of new classes of antimalarial drug candidates. A new class of antimalarial drugs, preferably with a novel mechanism of action that could circumvent the current resistance profile of *Plasmodium* (the causative organism of malaria) is highly desirable and needed. Since the molecular scaffolds as well as the pharmacophore features of a compound define the uniqueness of a compound, exploration of scaffolds of NAA may lead to identification of new antimalarial chemotypes. The term molecular scaffold is used to describe the core structure of a molecule and it determines the spatial orientation within the binding pocket of biological targets [9]. Hence, compounds with similar pharmacophore features but different scaffolds may have different bioactivity or mechanism of action (MOA).

It is believed that natural products are a good source of novel molecular scaffolds [10,11,12,13,14,15] and scaffolds derived from natural compounds have preferable or privileged scaffold architectures [16]. Since the scaffolds of natural compounds are potentially valuable, assessing the scaffold diversity of NAA and comparing them to CRAD and MMV is a logical starting point for identification of scaffolds that are meaningful for antimalarial drug design/discovery. Such scaffolds may be starting points for new classes of antimalarial drug candidates.

**Figure 1:**
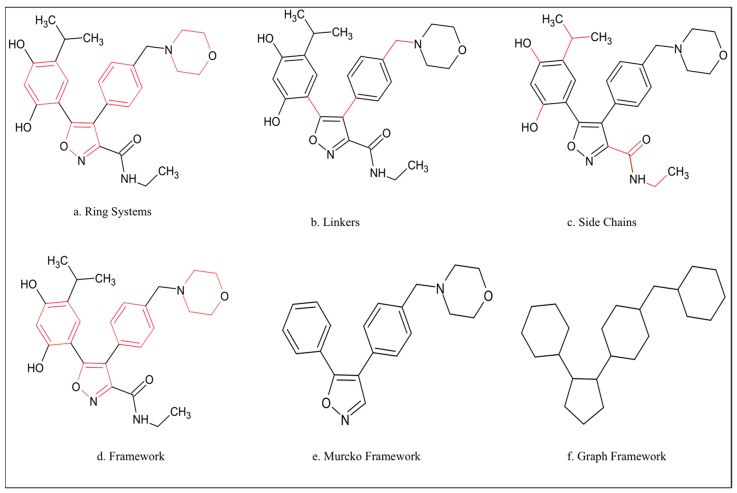
The framework representations of an exemplar molecular structure.

Scaffold diversity is one of many parameters that may be used to characterize compound libraries [17] and assess chemical diversity based on the scaffolds and ring systems in structures [1,18,19,20,21]. In order to analyze the scaffold diversity of a compound library, a suitable representation of a scaffold is needed. One of these representations is the Murcko framework/scaffold, proposed by Bemis and Murcko [22], which has been used to analyze the structures of known drugs and identify the common features in screening libraries and natural products [14,23]. The Murcko framework (Figure 1e) of a structure consists of all the ring systems and all the linkers that connect the ring systems [24]. This framework is obtained by pruning all side-chain atoms, *i.e.*, non-ring atoms not on a direct path between two ring systems (Figure 1) [1,24]. The method separates molecules into ring systems (Figure 1a), linkers (Figure 1b), side chain atoms (Figure 1c), and the framework (Figure 1d), which is the union of ring systems and linkers in a molecule. A Murcko framework (Figure 1e) keeps information on atom type, whereas a graph framework (Figure 1f) reduces all atoms to carbon and all bonds to single bonds. The Murcko framework defines only molecular topology and contains no three-dimensional or stereochemical information [1]. The frequency of Murcko frameworks or scaffolds have been used to define the structural diversity of chemical databases [1].

A number of studies have been carried out to explore the scaffold diversity of compound sets based upon the frequency of Murcko scaffolds and to identify unique scaffolds. For example, Bemis and Murcko used the Murcko scaffold to establish the scaffold diversity of 5129 known drugs and identified 1179 scaffolds [22]. An analogous analysis of the Chemical Abstracts Service (CAS) Registry of over million compounds also indicated that a large percentage of organic compounds are embodied by only a small percentage of scaffolds [25]. An analogous conclusion was drawn from a study that looked at the co-occurrence of scaffolds in a chemical database [26]. The study found that specific scaffolds and combinations of scaffolds were far more frequent than others and were therefore termed “Chemical Clichs” [26]. In a study that explored scaffolds present in a set of approximately 150,000 bioactive compounds, only 780 simple aromatic scaffolds were uncovered [19]. In another study, analysis of scaffold diversity of seven representative commercial and proprietary compound libraries revealed only a small number of well represented scaffolds and a high percentage of singleton scaffolds [24]. With specific reference to natural products, Grabowski and Schneider [14] have reviewed structural diversity of molecular frameworks and scaffolds of collections of drugs and drug-like molecules, pure natural products, and natural product derived compounds. Among the natural product library, they identified more than one thousand scaffolds that were not contained in any other compound set analyzed [27]. Although Lee and Schneider [10] demonstrated, using self-organizing maps (SOM), that current trade drugs and natural products have several topological pharmacophore patterns in common, they showed that only 17% of the scaffolds found in natural products could be found in registered drugs [10].

Another way to analyze the scaffold diversity of a compound library is with Scaffold Trees. Wetzel *et al.* [28] used the Scaffold Tree technique for chemoinformatic analysis of natural products. The technique iteratively removes rings one by one according to a set of prioritization rules, and in the end, the substructure with only one ring is obtained. Scaffold Hunter has been used to achieve this task for large datasets [29]. Scaffold Hunter, an interactive tool for intuitive hierarchical structuring, visualization and analysis of complex structure and bioactivity data, is used for navigation in and exploration of chemical space to identify “virtual scaffolds” that should share bioactivity properties with their parent compound or child scaffolds [29]. These virtual scaffolds are chemically meaningful entities that may provide opportunities for the identification of new biologically relevant scaffold classes [29].

The goal of this study was to use the methods described above to systematically identify and compare the scaffolds from NAA, CRAD and MMV. Specific objectives included analysis of the scaffold diversity of each dataset and exploration of similarity/diversity amongst scaffolds from NAA, CRAD and MMV. The relationship between the antiplasmodial activities and scaffold diversity of the NAA was explored by comparing the scaffold diversity of the bioactivity subgroups of NAA (*i.e.*, highly active (IC_50_ < 1 μm), active (IC_50_: 1–5 μm), moderately active (IC_50_: 5–10 μm) and low activity (IC_50_ > 10 μm)). From this study, we identified novel scaffolds in NAA that may be used as strategic and guiding frameworks for design of a natural product inspired antimalarial compound library. Interestingly, we observed greater scaffold diversity in the highly active subgroup of the NAA.

## 2. Results and Discussion

### 2.1. Scaffold Diversity

Scaffold counts and Cumulative Scaffold Frequency Plots (CSFP) were used to assess the scaffold diversity of three datasets: natural products with antiplasmodial activity (NAA), currently registered antimalarial drugs (CRAD) and malarial screen data from Medicines for Malaria Venture (MMV). In addition, the scaffold diversities of the highly active (HA), active (A), moderately active (MA) and low active (LA) subgroups of NAA were explored.

#### 2.1.1. Higher Scaffold Diversity Observed for the NAA Dataset

##### Scaffold Counts and Cumulative Scaffold Frequency Plots (CSFP)

Scaffold counts enumerate the number of compounds included in the analysis (M) and the number of scaffolds (N_s_) as well as the number of singleton scaffolds (N_ss_) generated from such compounds. With regards to the analysis of Level 1 scaffolds (from Scaffold Tree) for natural products with antiplasmodial activity (NAA), currently registered antimalarial drugs (CRAD) and malarial screen data from Medicines for Malaria Venture (MMV), Table 1 shows the ratios of scaffolds to molecules (N_s_/M), singleton scaffold to molecules (N_ss_/M) and singleton scaffolds to total scaffolds (N_ss_/N_s_). Higher values of these ratios are indicative of the greater scaffold diversity of the dataset. Applying these metrics to the Level 1 scaffolds, the MMV and NAA data sets showed a lower ratio of scaffolds to molecules (N_s_/M = 0.11 and 0.29, respectively) compared to CRAD (N_s_/M = 0.59). A similar trend was observed with the singleton scaffold to molecules (N_ss_/M). This indicates that MMV and NAA data sets contain heavily represented scaffolds. In other words, for MMV there are 10 molecules for every scaffold, 3.3 molecules for every scaffold in NAA while for CRAD there are 1.8 molecules for every scaffold. This result suggests that CRAD has the greatest scaffold diversity amongst these datasets. The inherent bias in this dataset is however that limited numbers of molecules from specific scaffolds are normally taken through the drug development pipeline.

**Table 1:** Parameters from the scaffold diversity analysis of CRAD, NAA and MMV (using Level 1 scaffolds) ^1^.

	N_s_/M	N_ss_/M	N_ss_/Ns	P25	P50	P75	AUC
Currently registered Antimalarial Drugs (CRAD)	0.59	0.48	0.81	6.47	17.97	49.91	6794
Natural products with *in vitro* antiplasmodial activity (NAA)	0.29	0.17	0.57	1.65	6.75	27.58	8017
Malarial screen data from Medicines for Malaria Venture (MMV)	0.11	0.05	0.53	0.11	1.02	9.39	9043

^1^ The parameters include ratios of scaffolds to molecules (**N_s_/M**), singleton scaffolds to molecule (**N_ss_/M**), singleton scaffolds to total scaffolds (**N_ss_/N_s_**), percentage of scaffolds that represent 25% of molecules (**P_25_**), percentage of scaffolds that represent 50% of molecules (**P_50_**), percentage of scaffolds that represent 75% of molecules (**P_75_**) and area under curve (**AUC**).

The ratio of singleton scaffolds to scaffolds (N_ss_/N_s_) provides more information on the distribution of molecules over scaffolds. For example, in CRAD with the highest ratio of scaffolds to molecules (N_s_/M = 0.59), suggesting that the data set has high scaffold diversity, the proportion of singleton scaffolds to scaffolds (N_ss_/N_s_) is 0.81; therefore, 81% of scaffolds (13 scaffolds) represent only 1 molecule each, and 19% of scaffolds (3 scaffolds) represent the remaining 14 molecules. This indicates that a large proportion of the Level 1 scaffolds in CRAD are singletons, suggesting that the distribution of molecules over scaffolds is uneven. In comparison, the NAA and MMV data sets showed lower ratio of singleton scaffolds to scaffolds (N_ss_/N_s_ = 0.57 and 0.53, respectively), indicating that a lower proportion of their Level 1 scaffolds are singletons and a more even distribution of molecules over scaffolds. This observation may be expected since NAA and MMV are screening collections containing a selection of antiplasmodial compounds that are equally represented.

Cumulative Scaffold Frequency Plots (CSFP) gives an indication of the distribution of compounds over the molecular scaffolds [24]. It was generated by plotting percentage of scaffolds against percentage of molecules as detailed in the experimental section. CSFP are interpreted thus: A diagonal line point to an equal distribution of compounds across the molecular scaffolds, while curves that are above the diagonal line (*i.e.*, with steeper gradients) represents compound datasets with low molecular scaffold diversity. The P_n_ values (*n* = 25%, 50% and 75%) and the area under the curve (AUC) are quantitative parameters estimated from the CSFP. The P_n_ values (where *n* is 25%, 50%, and 75%) indicate the percentage of scaffolds that represent “*n*” percent of compounds; thus, P_50_ is the percentage of scaffolds that represent 50% of all compounds in the dataset.

**Figure 2:**
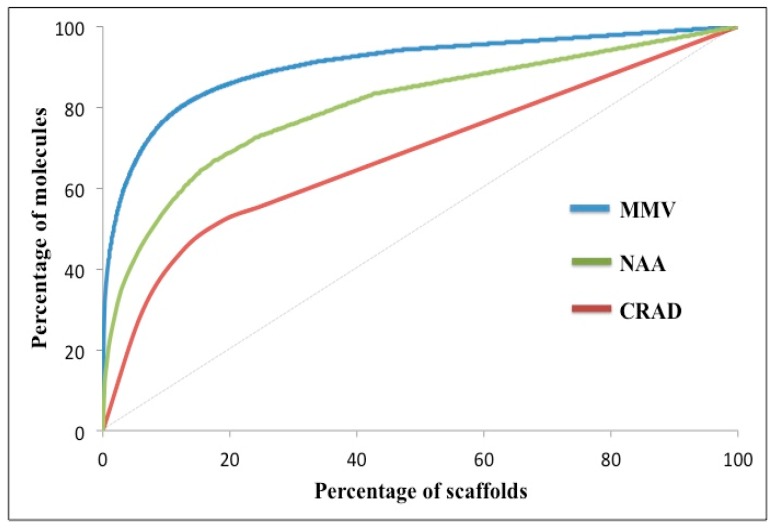
Cumulative Scaffold Frequency Plots of Murcko scaffolds from datasets. The plot is shown for currently registered antimalarial drugs (CRAD), natural products with *in vitro* antiplasmodial activity (NAA) and malarial screen data from Medicines for Malaria Venture (MMV). The closer the curve is to the diagonal line, the greater the scaffold diversity of the data set. Murcko scaffolds from CRAD showed the highest scaffold diversity.

The Cumulative Scaffold Frequency Plots (CSFP) of the Level 1 scaffolds for NAA, CRAD and MMV are shown in Figure 2 while the P_n_ values (where *n* is 25%, 50%, and 75%) and the AUC are included in Table 1. In the extreme case of scaffold diversity, each molecule in the collection will have its own scaffold, the plot would be a diagonal line from [0%, 0%] to [100%, 100%]; therefore, the closer the curve is to the diagonal line, the greater the diversity of the dataset. The result (Figure 2) showed that CRAD was closest to the diagonal line, indicative of its relatively higher scaffold diversity. MMV dataset was furthest from the diagonal line hence had the least scaffold diversity. The CSFP for NAA was between that of CRAD and MMV suggesting that the NAA dataset had higher scaffold diversity than MMV but lower scaffold diversity than CRAD. Since CSFP gives an indication of the distribution of compounds over the molecular scaffolds [24], the result also attests that compounds from NAA are more evenly distributed over their scaffolds than compounds from MMV but less evenly distributed than compounds from CRAD. Another point of note is that all curves began with a very steep gradient; this indicates the presence of scaffolds that represent a large proportion of the dataset. The shallow region of the curve represents the high proportion of singleton scaffolds.

For example in the MMV and NAA data sets, which have much lower ratios of singleton scaffolds to overall scaffolds (N_ss_/N_s_ = 0.53 and 0.57 respectively) compared to CRAD (N_ss_/N_s_ = 0.81), the curves level off later than for the CRAD data sets due to the lower proportion of singletons in the datasets. In order to compare the CSFP quantitatively, the P_n_ values (*n* = 25%, 50% and 75%) and the area under the curve (AUC) were estimated from the curves. P_n_ value is the percentage of scaffolds that represent “n” percent of compounds and larger P_n_ values indicate more scaffold diversity. From Table 1 it is clear that the P_25_, P_50_, and P_75_ of NAA (1.65, 6.75 and 27.58 respectively) were higher than those of MMV but lower than CRAD. This attests our earlier conclusions that NAA had greater scaffold diversity than MMV but lower than CRAD. The area under the CSFP curve (AUC) would be 5000 for a diagonal plot (*i.e.*, extreme scaffold diversity: each molecule in the collection will have its own scaffold). Thus, the lower the AUC, the greater the scaffold diversity or more even scaffold distribution of the dataset. The values of the AUC for NAA, CRAD and MMV are in line with the observed scaffold counts (N_s_/M) and P_n_.

In all, the proportion of scaffolds present in a dataset (N_s_/M), proportion of singleton scaffolds (N_ss_/N_s_) and the Cumulative Scaffold Frequency Plots (CSFP) were useful indicators of the scaffold diversity of the datasets studied. The greatest scaffold diversity was observed in CRAD. This result is expected, as mentioned earlier, since the CRAD dataset represents selected compounds that came from diverse chemical screening libraries during drug development and have successfully made it to market as antimalarial drugs. A more interesting conclusion from these results is that scaffolds from NAA (a collection of only natural compounds active against *Plasmodium*) were more diverse than those from MMV (a collection of synthetic and natural compounds active against *Plasmodium*). The lower scaffold diversity observed for MMV may be attributed to the synthetic compounds, which have low scaffold diversity, within this collection of compounds [30]. The low scaffold diversity of synthetic compounds stems from the nature of medicinal chemistry research over the past few decades, which has focused upon familiar and limited sets of biological targets as well as feasible chemical synthesis [30]. Consequently, these synthetic compounds may be intrinsically biased towards feasible chemical synthetic pathways and known bioactivity chemical space.

Scaffold diversity has been reported to be most crucial to the functional diversity of a collection of compounds. Although functional group substituents are important for *interaction* or binding with macromolecules, the central scaffold of a compound determines its spatial orientation and shape-space coverage [30]. Therefore, the higher scaffold diversity of NAA over MMV suggests that NAA may cover a wider biological chemical space than MMV. The potential to cover uncharted regions of the antimalarial chemical space increases the prospect of discovering potential antimalarial drugs with novel mechanism of antimalarial bioactivity. In conclusion, the higher scaffold diversity of NAA thus increases the likelihood to detect hit compounds with novel mechanism of action.

#### 2.1.2. Bioactivity Subgroups of NAA (HA, A, MA and LA)

##### Scaffold Counts and Cumulative Scaffold Frequency Plots (CSFP)

The scaffold count parameters obtained for the comparison of the subgroups of NAA (highly active (HA), active (A), moderately active (MA) and low active (LA)), using Murcko scaffolds, are outlined in Table 2. Though HA had the highest proportion of scaffolds to molecules (N_s_/M = 0.66), there was no significant difference amongst the N_s_/M ratios of HA, A (0.60) and MA (0.64) except for LA (0.52) (*p* = 0.001). The ratio of singleton scaffolds to total scaffolds (N_ss_/N_s_) was between 0.6 and 0.75 for the subgroups of NAA. This indicates that a high proportion of scaffolds were singletons and were not uniformly distributed across the HA, A, MA and LA datasets.

**Table 2:** Parameters from the scaffold diversity analysis of CRAD and the subgroups of NAA (HA, A, MA and LA) (using Murcko scaffolds).

	N_s_/M	N_ss_/M	N_ss_/N_s_	P_25_	P_50_	P_75_	AUC
CRAD	0.70	0.55	0.79	10.22	24.57	59.03	6247.6
HA	0.66	0.49	0.74	7.35	20.71	58.31	6410.2
A	0.60	0.40	0.67	6.59	18.96	54.50	6588.4
MA	0.64	0.49	0.77	6.16	18.53	55.68	6577.8
LA	0.52	0.38	0.73	4.19	13.96	46.50	7026.2

The parameters include ratios of scaffolds to molecules (**N_s_/M**), singleton scaffolds to molecule (**N_ss_/M**), singleton scaffolds to total scaffolds (**N_ss_/N_s_**); percentage of scaffolds that represent 25% of molecules (**P_25_**), percentage of scaffolds that represent 50% of molecules (**P_50_**), percentage of scaffolds that represent 75% of molecules (**P_75_**) and area under curve (**AUC**) for analysis of CRAD and the subgroups of NAA.

**Figure 3:**
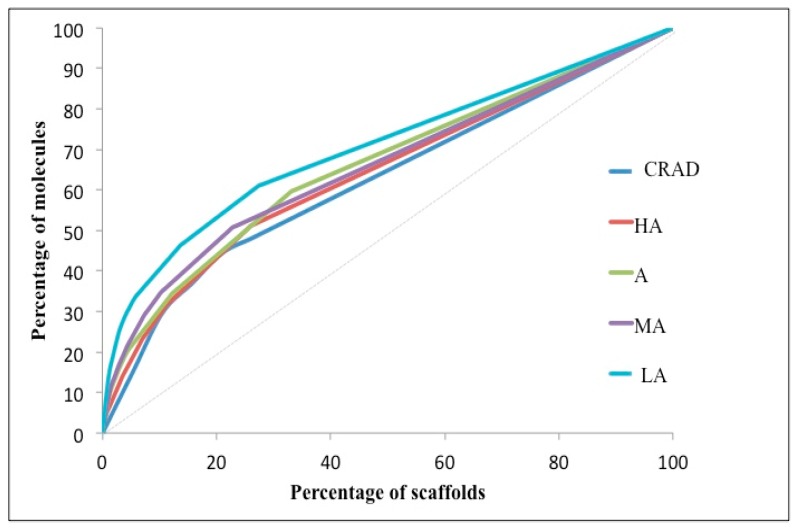
Cumulative Scaffold Frequency Plots of Level 1 scaffold of datasets. Plots for currently registered antimalarial drugs (CRAD), natural products with *in vitro* antiplasmodial activity (NAA) subgroups: HA-highly active, A-active, MA-moderately active and LA-low active are shown. The closer the curve is to the diagonal line, the greater the scaffold diversity of the data set. The low active NAA clearly has the least scaffold diversity.

Figure 3 shows the Cumulative Scaffold Frequency Plots (CSFP) for the Murcko scaffolds of the subgroups of NAA (HA, A, MA and LA) and CRAD (as a locus). As expected, the plot for CRAD was closest to the diagonal due to its high scaffold diversity. Amongst the subgroups of NAA, the low active (LA) subgroup of the NAA data set was farthest from the diagonal line, indicating that it had the lowest scaffold diversity. There was an overlap of the CSFP of the other subgroups of NAA (HA, A and MA). All the CSFPs level off early due to the high proportion of singletons in the datasets. To decipher the order of scaffold diversity amongst the subgroups of NAA (HA, A and MA), the P_n_ and AUC of the CSFP were estimated (Table 2). The larger the P_n_ values, the greater the scaffold diversity and the lower the AUC, the greater the scaffold diversity. Amongst the subgroups of NAA, the P_25_, P_50_ and P_75_ values for HA, A and MA were significantly higher than the LA. This suggests greater scaffold diversity in HA, A and MA over LA. The AUC values for the subgroups of NAA followed a similar trend as observed for the P_n_ values though in the reverse order; the smallest AUC was observed in HA and the highest AUC in LA. In general, these results indicate the apparent scaffold diversity amongst the activity subgroups of NAA and revealed an apparent correlation between scaffold diversity and antiplasmodial activities (*i.e.*, the greater the scaffold diversity, the broader the antiplasmodial activities). Greater scaffold diversity suggests better coverage of chemical space and thus increases the chance of finding compounds that interact with more biological targets to elicit bioactivity. The relationship between scaffold diversity and the antiplasmodial activity of NAA is thus clear.

### 2.2. Molecular Similarity of Scaffolds Reveal Differences between the NAA and CRAD Datasets

In the quest to find unique scaffolds that are not present in currently registered antimalarial drugs, we assessed the molecular similarity between the scaffolds obtained from currently approved antimalarial drugs (CRAD), natural products with antimalarial activities (NAA) and the malarial screen library from Medicines for Malaria Venture (MMV). We also took a closer look at the molecular similarity between the scaffolds from CRAD and bioactivity subgroups of NAA (highly-HA, active-A, moderately active-MA and low active-LA).

**Figure 4:**
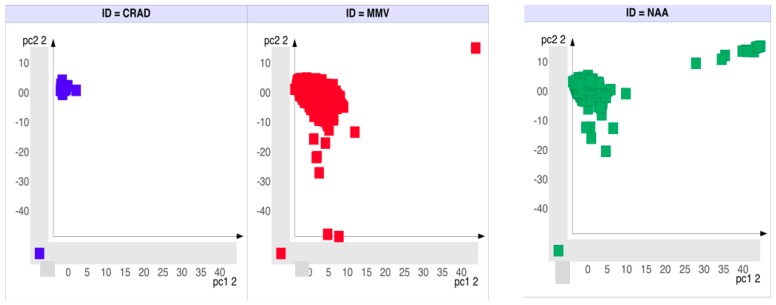
Scaffold structural similarity assessment using principal components analysis. Structural similarity between Level 1 scaffolds of natural products with antiplasmodial activities (NAA) and public malarial screen data (MMV) were assessed. “Skelspheres descriptors” from Datawarrior were used to assess structural similarity and generate principal components. The 3D plot of the first three principal components showed that most of the scaffolds from NAA occupy the same chemical space as scaffolds from MMV. However there were NAA scaffolds that were outliers from the main cluster.

Principal component analysis (PCA) was done to assess the structural similarity amongst the scaffolds from CRAD, NAA and MMV based on chemical bits-fingerprints. The results (Figure 4) revealed that though most of the scaffolds from NAA occupy the same chemical space as scaffolds from MMV, there were NAA scaffolds that were outliers from the main cluster. In comparison to scaffolds from CRAD, the majority of the scaffolds from NAA occupied distinct regions of the molecular similarity space. These scaffolds from NAA are unique and are not present in scaffolds from CRAD. The MMV also contain scaffolds that are located in different regions of the chemical space, suggesting that they are not present within the CRAD. Figure S1 contains a list of the NAA scaffolds that are not present in CRAD.

**Figure 5:**
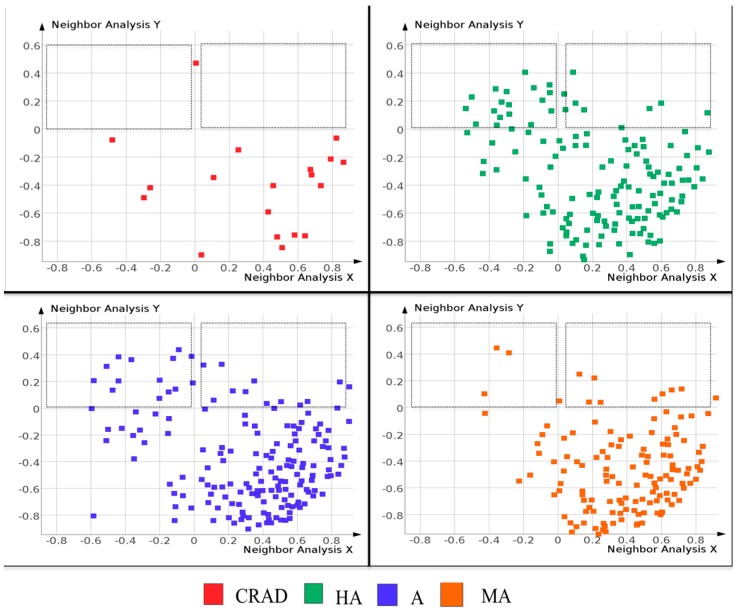
Scaffold structural similarity using Self-Organizing Maps (SOM). Shown above are the self-organizing maps (SOM) of currently registered antimalarial drugs (CRAD) and bioactivity subgroups of natural products with *in vitro* antiplasmodial activity (NAA): HA-highly active, A-active and MA-moderately active. Markers represent scaffolds that are arranged in a two-dimensional space based on scaffold structural similarity. The individual SOM clearly showed that scaffolds from the NAA (HA, A and MA) occupied distinct regions of the SOM (in the upper quadrants and marked with black rectangles) where scaffolds from CRAD are not found.

A Self-Organizing Map (SOM) (Figure 5) was used to arrange scaffolds in a two dimensional space in order to visualize similarity between CRAD and bioactivity subgroups of NAA (HA, A and MA) as well as to identify unique scaffolds in these bioactivity subgroups. The SOM (Figure 5) showed that some scaffolds from the bioactivity subgroups of NAA (HA, A and MA) occupied distinct regions of the map where scaffolds from CRAD were not found. This suggests that these scaffolds are not similar to the scaffolds from CRAD and thus unique to the dataset. 

**Figure 6:**
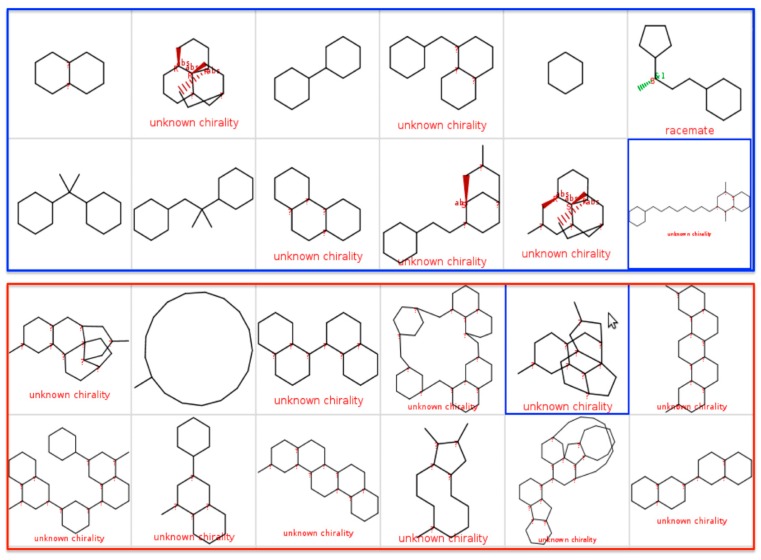
Comparison of top twelve scaffolds from the CRAD and NAA datasets. The top twelve Murcko scaffolds from currently registered antimalarial drugs (CRAD) are shown in the top blue panel. The top twelve Murcko scaffolds from the highly active natural products with *in vitro* antiplasmodial activity (HA) are shown in the bottom red panel. There were no similar scaffolds between the two datasets.

**Figure 7:**
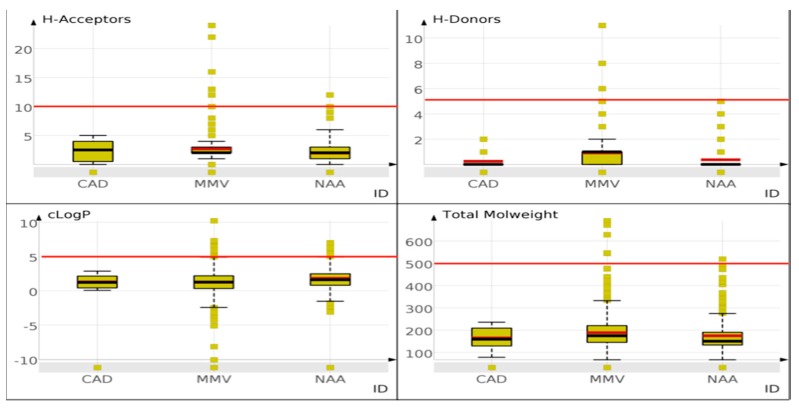
Drug-like properties of scaffolds from the datasets analysed. Properties like number of hydrogen bond donors and acceptors, cLogP and molecular weight of scaffolds from CRAD, NAA and MMV were assessed.

Evaluation of the top twelve scaffolds (based on frequency of occurrence) from CRAD and HA (Figure 6) revealed no similar scaffolds between these two scaffold sets. In all, given that unique scaffolds may have unique orientation within drug targets [9] and thus unique mechanism of action, these scaffolds may be explored as new frameworks for antimalarial drug design. In addition, Figure 7 showed that most of the scaffolds from NAA and MMV have drug-like properties (average hydrogen bond acceptors less than 5, average hydrogen bond donors less than 2, average calculated logarithm of partition coefficient less than 3 and average total molecular weight less than 200) that are far below the cut-off recommended by Lipinski’s models [31,32].

This “drug-likeness window” of scaffolds from NAA allows the addition of functional groups unto these scaffolds, during the design of potential natural product based antimalarial drugs, without exceeding the recommended drug-likeness cut-offs.

### 2.3. Scaffold Tree: Revealing Virtual Scaffolds

**Figure 8:**
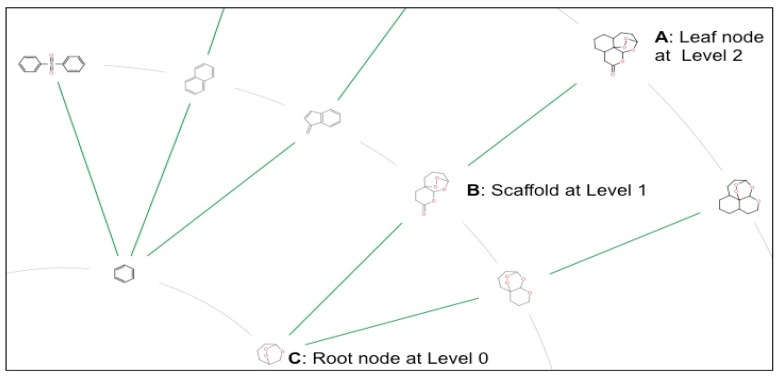
A section of a Scaffold Tree generated with Scaffold Hunter for currently registered antimalarial drugs (complete scaffold tree available in Figure S2). The technique first prunes all terminal side chains from parent molecules (**A**); then iteratively removes rings one by one according to a set of prioritization rules from the parent molecule (A) to get the intermediate scaffold (**B**); At the end, the scaffold with only one ring (**C**) is obtained. Scaffolds B and C represent “virtual scaffolds” that may have bioactivities similar to or higher than the parent compound (**A**).

Scaffold Tree is a hierarchical classification of chemical scaffolds or ring systems from compounds obtained by stepwise pruning of all terminal side chains and rings (Figure 8). The Scaffold Trees for CRAD and subgroups of NAA (HA, A, MA and LA) datasets generated using Scaffold Hunter are provided as portable data format files in the Figures S2–S6. These Scaffold Trees consist of concentric circles with the parent compound and the scaffolds arranged along the outer and inner circles respectively.

The resulting Scaffold Trees of CRAD, A, MA and LA (Figures S2–S6) showed no scaffolds at Level 5, Level 9, Level 10 and Level 11 respectively. However, HA had scaffolds at all levels of the Scaffold Tree up to Level 11. The presence of scaffolds at increasing levels of the Scaffold Tree indicate the predominance of ring systems and increasing molecular complexity of the compounds in a dataset. These results therefore suggest that the NAA (HA, A, MA and LA) have greater molecular complexity and more ring systems, as previously reported for natural products [33]. In addition, the most active NAA *i.e.*, HA may contain compounds with high molecular complexity and number of ring systems.

In addition, navigation of the building “scaffold” of the compounds in the datasets through the Scaffold Tree enabled us to visualize “virtual scaffolds” that may share bioactivity properties with their parent compound or child scaffolds [29]. These virtual scaffolds represent previously unexamined molecules that may for example exhibit higher potency and thus provide opportunities to venture into uncharted biologically relevant chemical space. These scaffolds may be of particular interest as starting points for subsequent synthesis of the parent compounds.

In summary, the Scaffold Tree allowed the visualization of the complexity of the ring systems of the compounds in the datasets analyzed. In addition, the Scaffold Tree contains “virtual scaffolds” that are chemically meaningful entities and may provide new opportunities for the identification of new bioactive compounds.

## 3. Materials and Methods

### 3.1. Datasets for Scaffold Analysis

The scaffold analysis of three datasets were explored: natural products with *in vitro* antiplasmodial activities (NAA) compiled from literature, PhD and Masters theses and public chemical databases; currently registered antimalarial drugs (CRAD) from ChEMBL and malaria box from Medicines for Malaria Venture (MMV). In total, there were 1036, 27, and 18923 compounds in NAA, CRAD and MMV, respectively. Comparing a large collection of compounds to a small collection is not a concern because studies have shown that large combinatorial libraries may contain a very low proportion of scaffolds as compared to smaller collections [21,34,35]. The NAA was subdivided in four subgroups based on their antiplasmodial activities (IC_50_): highly active (HA) (IC_50_: <1 µM), active (A) (IC_50_: 1–5 µM), moderately active (MA) (IC_50_: 5–10 µM) and low activity (LA) (IC_50_: >10 µM).

### 3.2. Generation of Scaffolds and Scaffold Trees

Two scaffold representations were used to analyze the scaffold diversity of the three datasets: Murcko scaffolds [22] and the Level 1 scaffolds from a Scaffold Tree [28], both of which are suitable for compounds containing cyclic systems. Murcko scaffolds were generated using a Konstanz Information Miner (KNIME) workflow [36]. The file containing the smiles and identifiers for each dataset were read and converted to RDKit molecules [37]. The Murcko scaffolds were generated with the RDKit “find Murcko scaffolds” [37] and written to a comma separated version file.

Scaffold Hunter was used to reduce each molecule in the dataset to its scaffold and arrange the resulting scaffolds hierarchically in a Scaffold Tree [28]. Scaffold Trees (Figure 8) were generated for CRAD, NAA (and its subgroups) and MMV. The molecules in each dataset were represented as the leaf nodes of the Scaffold Tree. Lower levels of the Scaffold Tree were obtained by iterative removal of rings according to a default rule set that are designed to retain the most richly functionalized ring systems and are intended to be intuitive to a synthetic medicinal chemist [24]. This process continued until only one ring remains at the root node of the tree (Level 0). The subsequent levels or nodes in the tree are numbered starting from 1. Different molecules may have different levels, depending on the complexity or the number of rings in the studied molecules; hence the number of levels of the Scaffold Tree was counted for each dataset. The scaffolds at each level of the Scaffold Tree were also counted for each data set.

The Level 1 scaffolds from the Scaffold Tree were used to assess the scaffold diversity of the CRAD, NAA and MMV datasets because it has been reported to be most informative and appropriate for such analysis [24]. The scaffolds at Level 0 were not chosen because they are usually too simple to characterize the structural features of the studied molecules and at levels above Level 1, the scaffolds become more complicated and even identical to the Murcko framework. In addition, some compounds with low molecular weight or less molecular complexity usually do not have Level 3 or above. Murcko scaffolds have been reported to be informative for Structural Activity Relationships (SAR) [22] and was selected to evaluate the scaffold diversity and evaluate the association between scaffold diversity and *in vitro* antiplasmodial activities for subgroups of NAA (HA, A, MA and LA).

### 3.3. Scaffold Diversity Analysis

Scaffold diversity was investigated using two parameters: Scaffold counts and Cumulative Scaffold Frequency Plots (CSFP). These parameters provided information on the distribution of molecules over scaffolds.

#### 3.3.1. Scaffold Counts

The scaffold diversity analysis was performed on the CRAD, MMV, NAA and bioactivity subgroups of NAA (HA, A, MA and LA) data sets. The numbers of unique scaffolds for each data set were counted (N_s_) along with the number of molecules they represent (M); this is referred to as the scaffold frequency. The number of singleton scaffolds was also recorded (N_ss_); singleton scaffolds are scaffolds that are only present in one exemplar molecule. The ratio of scaffolds to compounds (N_s_/M) and the ratio of singleton scaffolds to all scaffolds (N_ss_/N_s_) were used to assess the diversity of scaffold space.

#### 3.3.2. Cumulative Scaffold Frequency Plots (CSFP)

To generate cumulative scaffold frequency plots (CSFP), the scaffolds were first sorted by their scaffold frequency (most frequent to least frequent). The cumulative percentage of scaffolds was then plotted against the cumulative scaffold frequency as a percentage of total molecules. CSFP were generated for CRAD, MMV and NAA using Level 1 scaffold representations. Murcko scaffolds were used to construct the CSFPs for CRAD and bioactivity subgroups of NAA (HA, A, MA and N). From the CSFPs, the percentages of scaffolds that represent “*n*” percent of compounds were determined (where *n* = 25%, 50% and 75%). The area under the curve (AUC) was also estimated for each CSFP. Both the P_50_ and AUC have been used as measures of scaffold diversity [24].

### 3.4. Molecular Similarity amongst Scaffolds

Self-Organizing Map [SOM], a robust artificial neural network algorithm, was used to organize Murcko scaffolds of CRAD and bioactivity subgroups of NAA (HA, A, MA and LA) based on scaffold structural similarity. Scaffold structural similarity was assessed with “Fragfp descriptors” from DataWarrior [38]. Fragfp descriptors are substructure fragment dictionary based binary fingerprints similar to MDL keys [39]. Principal component analysis [PCA] was used to assess the structural similarity between Level 1 scaffolds from NAA and MMV. PCA was done on “Skelspheres descriptors” generated for the NAA and MMV scaffolds with DataWarrior. “Skelspheres descriptors” encodes circular spheres of atoms and bonds into a hashed binary fingerprint of 512 bits. “Skelspheres descriptors” also account for stereochemistry, counts duplicate fragments and encodes heteroatom-depleted skeletons. The first three principal components from the PCA were plotted in a three dimensional graph. Scaffolds from NAA (HA, A, MA and LA) that are not similar to scaffolds from CRAD may be novel in the antimalarial drug chemical space.

## 4. Conclusions

Murcko scaffolds and Scaffold Trees were generated from natural products with *in vitro* antiplasmodial activities (NAA); currently registered antimalarial drugs (CRAD) and malaria box from Medicine for Malaria ventures (MMV). The scaffold diversity of these antimalarial compound datasets were computed and compared. The scaffold count and cumulative scaffold frequency plots (CSFP) showed that CRAD is the most scaffold diverse dataset while NAA displayed more scaffold diversity than MMV. Amongst the bioactivity subgroups of NAA, the high active (HA) compound set had the highest scaffold diversity. The Scaffold count and Cumulative scaffold frequency plots (CSFP) were useful indicators of scaffold diversity of the antimalarial and antiplasmodial datasets studied.

It was evident that many of the scaffolds from the NAA (HA, A, MA and LA) were not similar to those from CRAD, thereby highlighting the novelty of these scaffolds in the antimalarial chemical space. Moreover, most of the scaffolds have desirable drug-like properties and these novel scaffolds may be used as frameworks to design new antimalarial focused compound libraries.

Scaffold Tree was also used to explore the scaffolds present in CRAD and bioactivity subgroups of NAA (HA, A, MA and LA). The presence of scaffolds at increasing levels of the Scaffold Tree for HA, A, MA and LA indicate the prevalence of ring systems and increasing molecular complexity of the compounds in these datasets. More importantly, virtual scaffolds present at different levels of the Scaffold Tree of the NAA compounds were identified, which are chemically significant, and may also provide starting points for new potential antimalarial compounds.

Overall, the exploration and comparison of the scaffolds from CRAD, MMV and NAA (HA, A, MA and LA) enabled us to find novel scaffolds and chemotypes that may result in progress towards design of new compound libraries and development drug candidates to combat malaria. This study also underscores the potentially significant contributions from nature to antimalarial drug development.

## Supplementary Materials

Supplementary materials can be accessed at: http://www.mdpi.com/1420-3049/21/1/104/s1.
