# Supplementary material for: Exploration of Scaffolds from Natural Products with Antiplasmodial Activities, Currently Registered Antimalarial Drugs and Public Malarial Screen Data
Source: Molecules. 2016 Jan 16;21(1):104. doi: 10.3390/molecules21010104 (PMC6273396; doi:10.3390/molecules21010104)
Supplement: Supplementary file 1 [file molecules-21-00104-s001.pdf]

Supplementary Materials: Exploration of Scaffolds from Natural Products with Antiplasmodial Activities, Currently Registered Antimalarial Drugs and Public Malarial Screen Data

Samuel Egieyeh, James Syce, Alan Christoffels and Sarel F. Malan

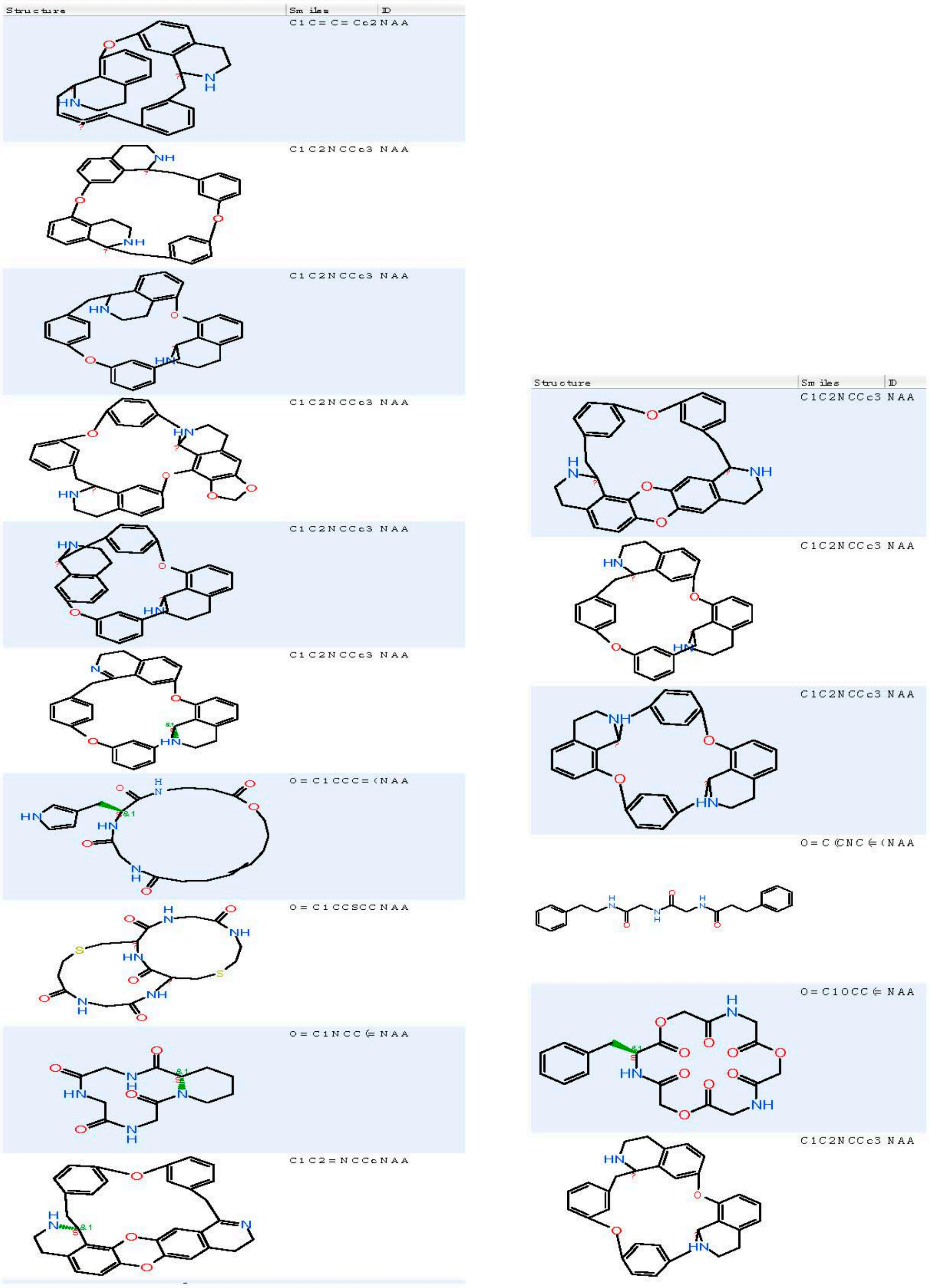

Figure S1. Unique Murcko scaffolds from natural products with in-vitro antiplasmodial activities.

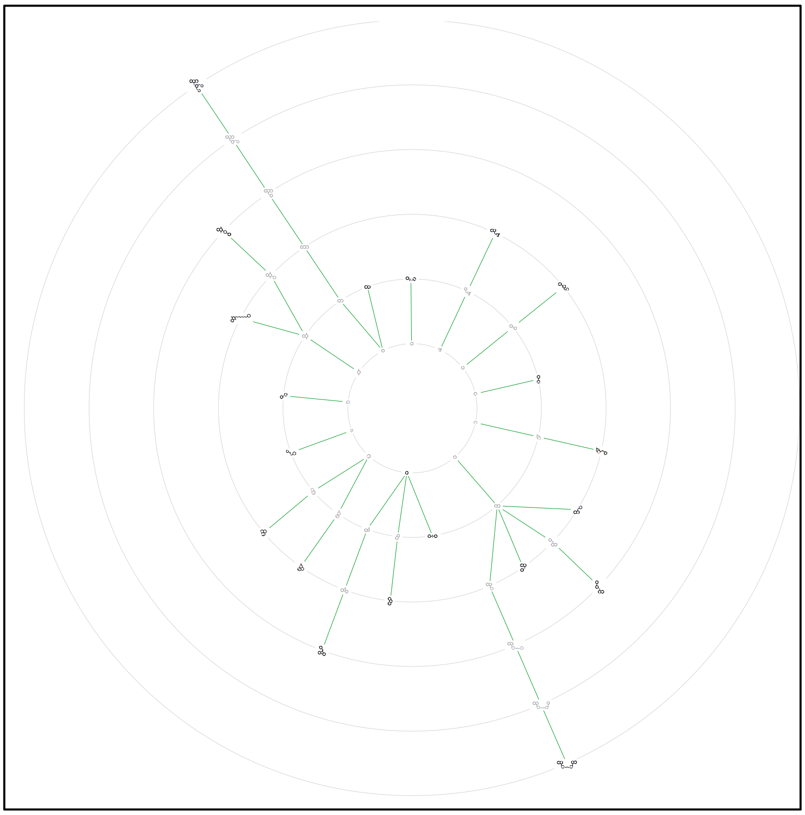

Figure S2. Scaffold Tree of currently registered antimalarial drugs (CRAD). The Scaffold Tree is between Level 0 to Level 5.

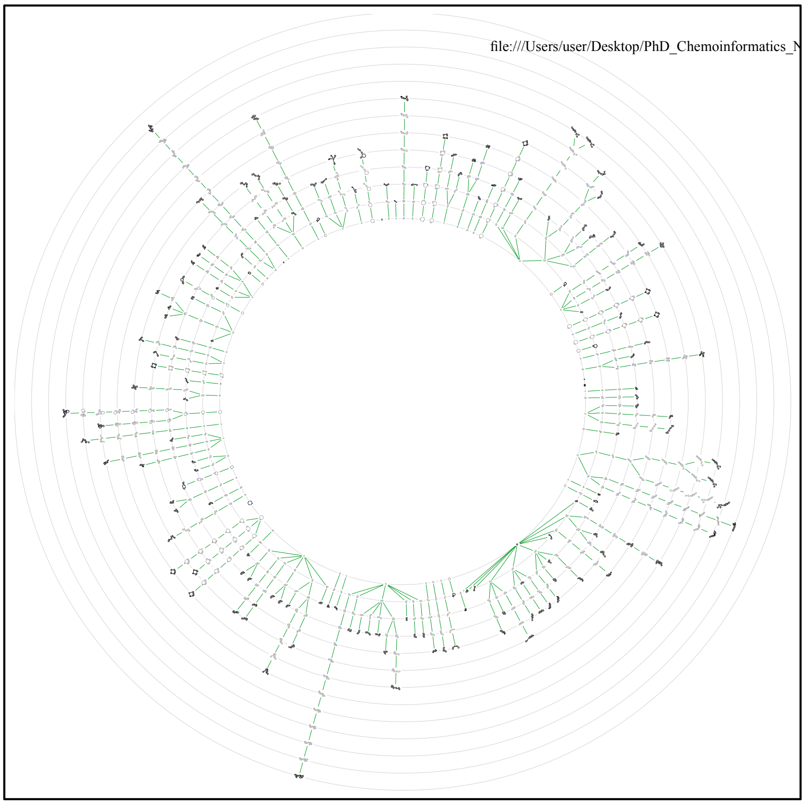

Figure S3. Scaffold Tree of highly active natural products with *in-vitro* antiplasmodial activities (HA). The Scaffold Tree is between Level 0 to Level 12.

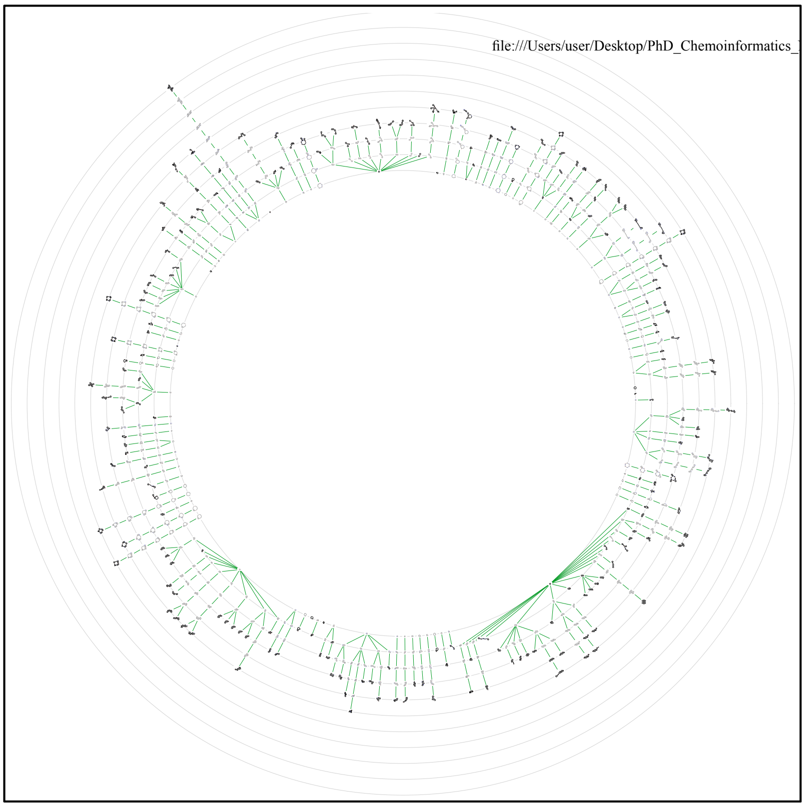

Figure S4. Scaffold Tree of active natural products with in-vitro antiplasmodial activities (A). The Scaffold Tree is between Level 0 to Level 10.

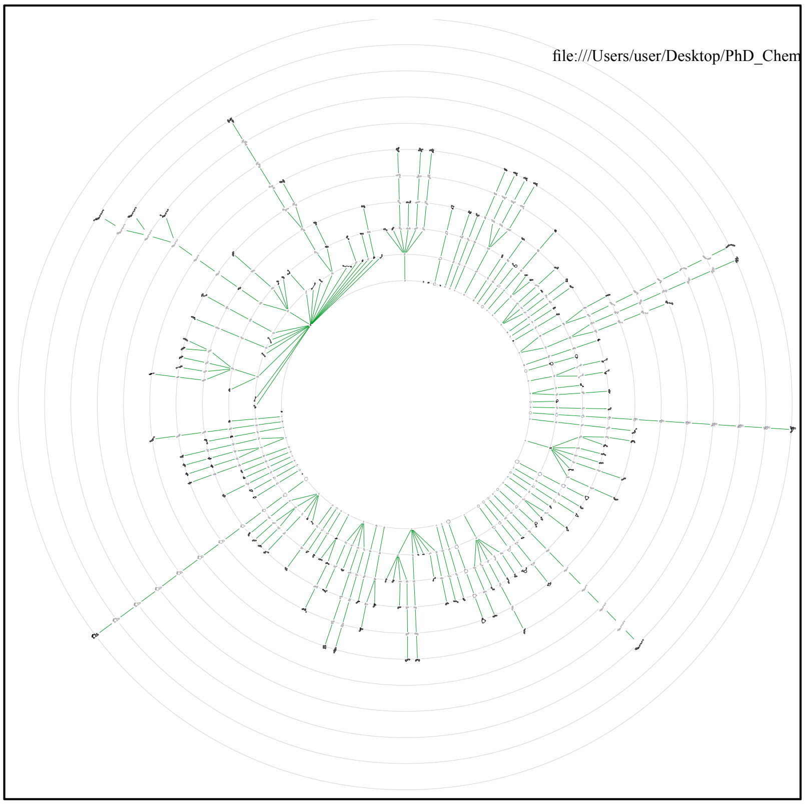

Figure S5. Scaffold Tree of moderately active natural products with in-vitro antiplasmodial activities (MA). The Scaffold Tree is between Level 0 to Level 10.

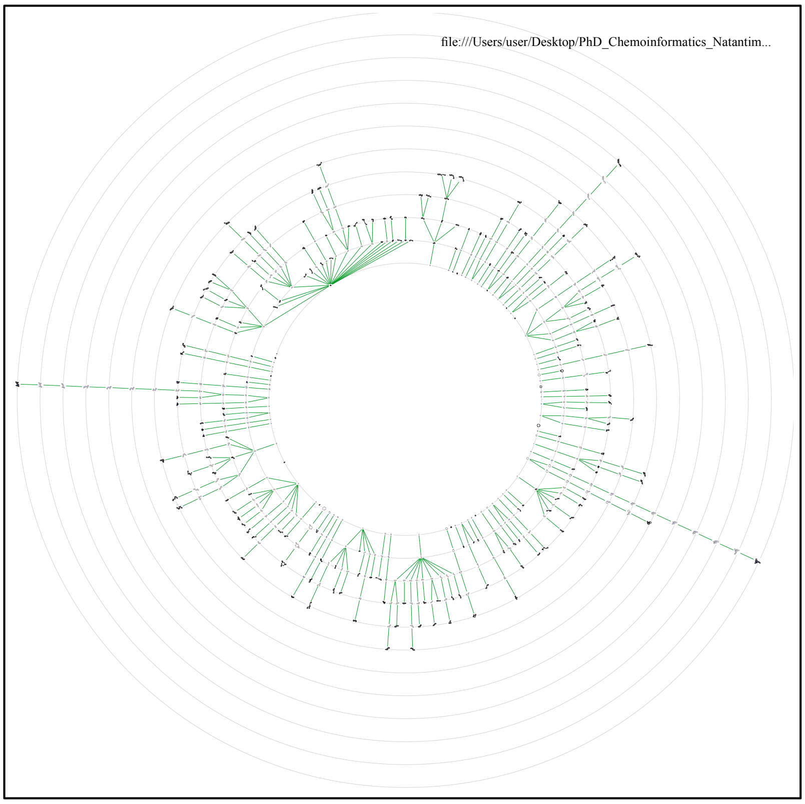

Figure S6. Scaffold Tree of lowly active natural products with in-vitro antiplasmodial activities (LA). The Scaffold Tree is between Level 0 to Level 11.
